# Effects of Resistance Training on Academic Outcomes in School-Aged Youth: A Systematic Review and Meta-Analysis

**DOI:** 10.1007/s40279-023-01881-6

**Published:** 2023-07-19

**Authors:** Katie Robinson, Nicholas Riley, Katherine Owen, Ryan Drew, Myrto F. Mavilidi, Charles H. Hillman, Avery D. Faigenbaum, Antonio Garcia-Hermoso, David Revalds Lubans

**Affiliations:** 1 Centre for Active Living and Learning, College of Human and Social Futures, University of Newcastle, Callaghan Campus, Callaghan, NSW 2308 Australia; 2 Hunter Medical Research Institute (HMRI), New Lambton, NSW Australia; 3 Prevention Research Collaboration, Sydney School of Public Health, The University of Sydney, Sydney, NSW Australia; 4 School of Environmental and Life Sciences, College of Engineering, Science and Environment, Newcastle, NSW Australia; 5 School of Education/Early Start, University of Wollongong, Wollongong, NSW Australia; 6 Illawarra Health and Medical Research Institute (IHMRI), Keiraville, Australia; 7 Department of Psychology, Northeastern University, Boston, MA USA; 8 Department of Physical Therapy, Movement and Rehabilitation Sciences, Northeastern University, Boston, MA USA; 9 Department of Kinesiology and Health Sciences, The College of New Jersey, Ewing, NJ 08628 USA; 10 Navarrabiomed, Hospital Universitario de Navarra (HUN), Universidad Pública de Navarra (UPNA), IdiSNA, Pamplona, Navarra Spain; 11 Faculty of Sport and Health Sciences, University of Jyväskylä, Jyväskylä, Finland

## Abstract

**Background:**

The primary aim of our systematic review and meta-analysis was to investigate the effect of resistance training on academic outcomes in school-aged youth.

**Methods:**

We conducted a systematic search of six electronic databases (CINAHL Complete, PsycINFO, SCOPUS, Ovid MEDLINE, SPORTDiscus and EMBASE) with no date restrictions. Studies were eligible if they: (a) included school-aged youth (5–18 years), and (b) examined the effect of resistance training on academic outcomes (i.e., cognitive function, academic achievement, and/or on-task behaviour in the classroom). Risk of bias was assessed using the appropriate Cochrane Risk of Bias Tools, funnel plots and Egger’s regression asymmetry tests. A structural equation modelling approach was used to conduct the meta-analysis.

**Results:**

Fifty-three studies were included in our systematic review. Participation in resistance training (ten studies with 53 effect sizes) had a small positive effect on the overall cognitive, academic and on-task behaviours in school-aged youth (standardized mean difference (SMD) 0.19, 95% confidence interval (CI) 0.05–0.32). Resistance training was more effective (SMD 0.26, 95% CI 0.10–0.42) than concurrent training, i.e., the combination of resistance training and aerobic training (SMD 0.11, 95% CI − 0.05–0.28). An additional 43 studies (including 211 effect sizes) examined the association between muscular fitness and cognition or academic achievement, also yielding a positive relationship (SMD 0.13, 95% CI 0.10–0.16).

**Conclusion:**

This review provides preliminary evidence that resistance training may improve cognitive function, academic performance, and on-task behaviours in school-aged youth.

**PROSPERO Registration:**

CRD42020175695.

**Supplementary Information:**

The online version contains supplementary material available at 10.1007/s40279-023-01881-6.

## Key Points

**Table:** 

Resistance training interventions had a small positive effect on the combined outcomes of cognition, academic achievement, and on-task behaviour in school-aged youth.
Resistance training was more effective than concurrent training.
Higher levels of muscular fitness were associated with greater performance in tests of cognition and academic achievement in school-aged youth.

## Introduction

International guidelines recommend children and adolescents participate in an average of 60 min of moderate to vigorous physical activity each day [1]. Further, it is advised that young people engage in muscle-strengthening activities at least 3 days per week [1, 2]. Resistance training is a specialized form of muscle strengthening activity designed to enhance muscular strength, local muscular endurance, and muscular power (hereafter the combined terms are referred to as muscular fitness) [3]. It involves the use of different modes of training with a variety of resistance loads, including but not limited to body weight, free weights, elastic bands, medicine balls, and kettlebells.

The benefits of resistance training for young people’s health and sport performance are well established: for example, resistance training can enhance performance through increased muscular fitness and positively contribute to improvements in motor skill performance, speed and power [3]. Adequate muscular fitness can also reduce the risk of injury and assist in rehabilitation [3]. In addition, health benefits such as improved cardiovascular fitness and body composition provide strong evidence for the promotion of resistance training for school-aged youth [3]. Global self-esteem and physical self-worth are two further psychological health outcomes positively impacted by resistance training [4]. While the benefits of resistance training are numerous, a significant proportion of youth are not meeting current guidelines for muscle-strengthening activity [5].

Cognitive abilities, defined as the set of mental processes that contribute to perception, memory, intellect and action [6, 7], are strongly associated with academic skills and are critical for children's development [8]. The impact of resistance training on cognitive outcomes and academic achievement for youth has not been established; however, recent systematic reviews have shown positive findings in adults and older adults [9–12]. A recent meta-analysis conducted by Wilke and colleagues revealed that resistance training had a positive effect on global cognition (standardized mean difference (SMD) 0.56, 95% confidence interval (CI) 0.22–0.90, *p* = 0.004), with selectively varied effects for inhibitory control (SMD 0.73, 95% CI 0.21–1.26, *p* = 0.01) and cognitive flexibility (SMD 0.36, 95% CI 0.17–0.55, *p* = 0.004) [12]. There are a range of potential behavioural, psychosocial and neurobiological mechanisms that may explain the effects of physical activity, including resistance training, on cognitive function and academic outcomes in youth [7, 13, 14]. There is a growing body of evidence suggesting that participation in physical activity leads to changes in brain structure and function [15]. To our knowledge, no previous study has examined the mechanisms responsible for the effects of resistance training in youth. However, the self-regulation skills that are inherent in resistance training (e.g., the monitoring of load) is one plausible mechanism that warrants further investigation [14]. While there is emerging evidence for the benefits of resistance training for children and adolescents’ cognitive function [16, 17], no systematic review of the literature has been conducted. Similarly, there is increasing interest in examining the associations between muscular fitness, cognition, and academic achievement in young people. For example, a study by Cancela et al. [18] found that academic achievement was moderately associated with strength, and the work of Syvaoja et al. [19] offers further support, with a longitudinal study that positively associates muscular fitness and overall academic achievement.

Despite growing evidence for the benefits of resistance training and positive associations between muscular fitness, cognitive and academic outcomes in youth, the existing evidence has not been quantitatively synthesized. Therefore, the primary aim of our systematic review and meta-analysis was to investigate the effect of resistance training on academic outcomes (i.e., cognitive function, academic achievement, and on-task behaviors) in school-aged youth, and our secondary aim was to examine muscular fitness and its relationship with cognition and academic achievement in the same population.

## Methods

Our systematic review and meta-analysis protocol was registered with the Preferred Reporting Items for Systematic Review and Meta-Analysis Protocols (PROSPERO) on 28 April 2020 (CRD42020175695). The conduct and reporting of this review adhere to the PRISMA (Preferred Reporting Items for Systematic Reviews and Meta Analyses) and PERSiST (implementing PRISMA in Exercise, Rehabilitation, Sport medicine and SporTs science) guidelines [20, 21].

### Eligibility Criteria

To be included in this review, all studies were required to meet the following criteria (using PICOS/PECOS criteria [22]): (1) Participants: school-aged children and adolescents (mean age 5–18 years) who were attending a primary or secondary school, but not university or tertiary education. Studies needed to assess the whole population, not be limited to a subgroup. Some special populations were eligible for inclusion. For example, participants with physical disabilities, overweight and obesity, and those with diagnosed mental health disorders. (2) Intervention/exposure characteristics: studies that assessed the relationship between resistance training or muscular fitness and cognition and academic outcomes. We use the *phrase academic outcomes* to refer to academic grades, performance on standardized tests, and on-task behaviour in the classroom. Our review includes acute studies (i.e., single bout) designed to examine the immediate effect of resistance training on cognitive function, and chronic studies (i.e., long-term intervention); and classify the intervention as either resistance training only or concurrent training (e.g., the combined training of strength and aerobic activities) [23]. (3) Comparison: non-exercise control group. (4) Outcome: cognition outcomes (i.e., attention, inhibitory control, cognitive flexibility, working memory, planning, fluid intelligence) and/or academic achievement (e.g., maths, languages and combined scores) and/or on-task behaviour. (5) Study design: use an experimental, quasi-experimental, parallel group, cluster, single-group study design, cross-sectional or longitudinal study design.

### Search Strategy

Structured electronic searches were conducted in the following databases: CINAHL Complete, PsycINFO, SCOPUS, Ovid MEDLINE, SPORTDiscus and EMBASE. The final search was carried out by the first author on 24 October 2022 and included all years prior to the search date. The selected search terms encompassed a combination of keywords relating to (i) resistance training, muscular fitness, (ii) cognition, executive function, on-task behaviour, and (iii) age limits. The full search strategy is presented in Online Supplemental Material (OSM) Resource 1. Only peer-reviewed publications that were published in English were considered, and no additional filters were applied during the search. Finally, a manual search of full-text articles’ reference lists was conducted to identify any additional publications.

### Selection Processes

**Fig. 1:**
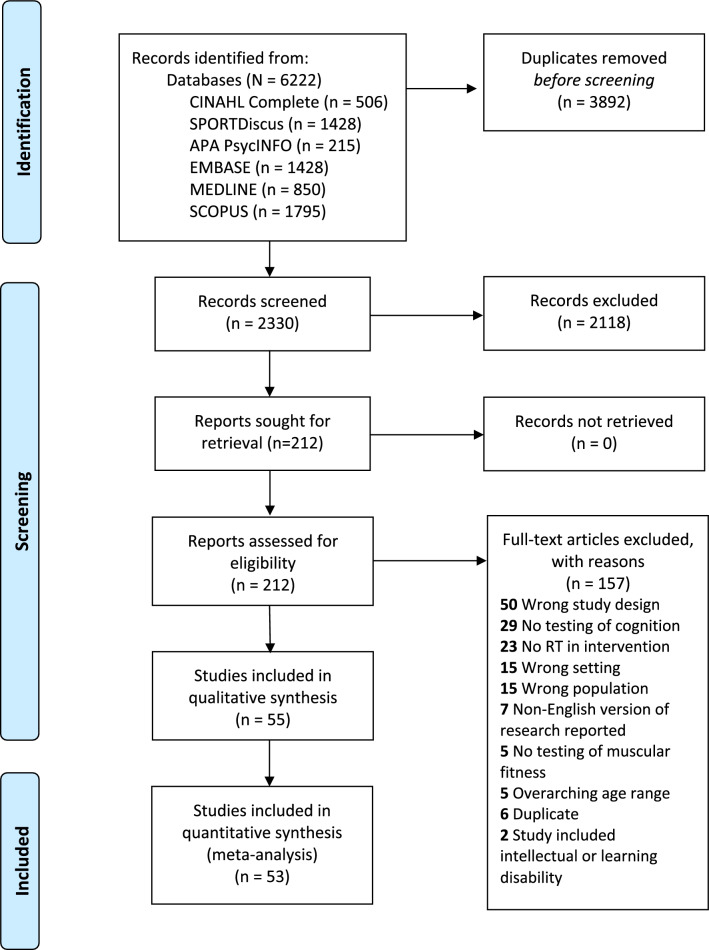
PRISMA flow diagram for study inclusion

First, two researchers independently screened titles and abstracts retrieved from databases and other sources for eligibility (Fig. 1). Next, relevant full texts were retrieved and independently screened by two researchers. All discrepancies regarding inclusion criteria fulfilment were resolved by a third researcher.

### Data Extraction

Two researchers independently extracted data from the eligible studies using a standardized extraction form. Key study characteristics were extracted and included: first author name, year of publication, study location (country), study design, sample size, sex and age of participants. Further characteristics were collected when investigating the primary outcome (resistance training studies) and included: assessment period (acute/chronic), study duration, intervention type, measure of cognition and academic outcomes (post-test mean scores of control and intervention groups). Regarding the secondary outcome, when cross-sectional and longitudinal studies reported the correlation between muscular fitness and cognition and academic outcomes, we also recorded if the result was adjusted for cardiorespiratory fitness.

Across all studies, when the relevant data were not reported in the study, we contacted the corresponding author and requested the additional information.

### Study Risk of Bias Assessment

An independent assessment of the risk of bias was conducted by two reviewers. Reviewers worked independently and met weekly to discuss score allocation. Discrepancies were discussed and were able to be resolved. A third reviewer although available was not required. The appropriate version of the Cochrane Risk of Bias Tool (RoB 2.0) was used to assess individually randomized and parallel trials or cluster randomized trials [24]. This tool assesses the risk of bias in the following domains: randomization process, deviation from intended intervention, missing data, measurement of outcome, and reporting [25]. The same two reviewers independently assessed the methodological quality of non-randomized studies by applying the 14 items of the National Institutes of Health (NIH) Quality Assessment Tool for Observational Cohort and Cross-Sectional Studies [26].

### Effect Measures

Summary measures included standardized mean differences, correlation coefficients, log odds ratios, and *F* values. For experimental studies, the effect size was calculated using post-test mean values and standard deviations from control and intervention groups [27]. Where multiple tests of cognition were conducted, each test score was extracted and treated individually. Where results were not reported adequately, we contacted the corresponding authors (one study was not included). All scores were converted to Cohen's *d* effect size (hereafter referred to as the SMD) and were defined as small (SMD: 0.20), medium (SMD: 0.50), and large (SMD: 0.80) [28]. We corrected Cohen’s *d* for sample size so that effect sizes for smaller studies were reduced to control for different sample sizes across studies [29].

We combined effect sizes using a structural equation modelling approach to multilevel meta-analysis. The main advantage of this approach is that it is not limited by the assumption of independence (i.e., effect sizes are nested within studies), and multiple effect sizes can be included from each study [30]. Unconditional mixed-effects models using maximum likelihood estimation were conducted to calculate the overall pooled effect size. For each pooled effect size, 95% likelihood-based CIs were calculated. All analyses were conducted using the metaSEM package [30] in R Version 4.0.2 [31].

The I^2^ statistic measures variability in the effect sizes (i.e., heterogeneity) [32]. An *I*^2^ statistic between 0 and 40% might not be important, 30–60% might represent moderate heterogeneity, 50–90% might represent substantial heterogeneity, and 75–100% considerable heterogeneity [33]. Heterogeneity was explored and explained using moderator analyses.

#### Primary Outcome: Moderators Examining the Effect of Resistance Training on Cognitive and Academic Outcomes

To provide future direction for how resistance training interventions can affect cognition, academic achievement, and on-task behaviour, we moderated by aspects of cognition. A broad approach was adopted to include all aspects of cognition and is presented in Table 1. Each outcome was broadly classified as:

*Cognition:* The set of mental processes that contribute to perception, memory, intellect and action [7].

*Academic achievement: *The extent to which a student has achieved their educational goals, commonly measured by examinations or continuous assessment (i.e., standardized tests, school grades) [7].

*On-task behaviour*: On-task behaviour (follows the class rules and is appropriate to the learning situation). Off-task behaviour (any behaviour that was not on-task and could be categorized as either motor off-task, noise off-task or passive off-task) [34].

**Table 1:** List of the measures used by the studies included in this review, organized by outcomes

Outcome	Measure
*Cognition*	
Attention	Simple Reaction Time, 4-Choice Reaction Time, d2 Test of Attention, Rapid Visual Information Processing (RVP) test, Finger tapping tests, modified Attention Network Test (ANT)
Cognitive flexibility	Trail making test (TMT), Dimensional Change Card Sort Test, Design Fluency Test, Digit symbol coding, verbal fluency test
Inhibition	Stroop test, modified Flanker task, Eriksen Flanker, Simon-task, Go/ no-go test, modified Attention Network Test (ANT)
Working memory	Serial n-back task, Spatial working memory test, Visual Memory-Wechsler Memory Scale, Modified delayed non‐matched‐to‐sample Task (DNMS), Forward memory span, Reverse memory span, Grid test, Forward and Backward digit span task
Planning	Zoo Map Test, modified Hanoi towers
Fluid intelligence	Woodcock-Muñoz test battery, D48 and Raven’s Progressive Matrices, Balance scale, Progressive matrices
*Academic achievement*	
Languages	School grades (assessed by teachers), Illinois Standardized Achievement Test (ISAT), Standardized test scores, Adapted standardized tests, Battery of General and Differential Aptitudes (BADyG E1)
Mathematics	School grades (assessed by teachers), 10-question math tests, Illinois Standardized Achievement Test (ISAT), Standardized test scores, Adapted standardized tests, Battery of General and Differential Aptitudes (BADyG E1)
Combined	School grades (assessed by teachers), academic questionnaires, Grade Point Average (GPA)
*On-task behaviour*	
On-task behaviour	Adapted momentary time sampling procedure

Note that many cognitive tests span multiple aspects of cognition, as indicated below

As there is some evidence that the effects of resistance training may differ by age and maturity status [3], we compared children (mean age 5–9 years) and adolescents (mean age 10–18 years). Next, we examined study characteristics to support future researchers in the decision making for the design of resistance-training interventions. Acute (single-session studies with immediate cognitive testing), compared to chronic interventions (studies $\ge$ 4 weeks with cognitive tests performed a minimum of 1 h post exercise), have previously been shown to effectively improve cognition, and comparing these results may direct future approaches for study duration and timing of cognitive tests [35]. Finally, we also compared the effects of resistance training and concurrent training programs [36].

#### Secondary Outcome: Moderators Examining the Association Between Muscular Fitness and Cognition and Academic Outcomes

The larger number of studies allowed for aspects of cognition to be analyzed by outcome: attention, inhibitory control, cognitive flexibility, working memory, planning, fluid intelligence (see Table 1). Also, we compared mathematics, languages and overall academic achievement based on current indications in this field [37]. School grades and standardized test results, although subject to methodological artifact, were combined in the analysis. Data that had been adjusted for cardiorespiratory fitness were compared. In many cases, additional adjustment for cardiorespiratory fitness weakens the association between muscular fitness and cognitive outcomes [38]. Consideration of participant characteristics analyzed age and study design moderators analyzed cross-sectional and longitudinal studies independently. Finally, to examine risk of bias within studies, we compared studies with high risk, some concerns and low risk of bias.

### Reporting Bias Across Studies

We assessed risk of bias across studies (publication bias) using funnel plots [39] and Egger’s regression asymmetry tests [40]. Effect sizes were plotted against the standard errors and then the symmetry was inspected. Next, we conducted Egger’s regression asymmetry tests by regressing the normalized effect estimate (effect size divided by its standard error) against precision (reciprocal of the standard error of the effect size). The regression line will pass through the origin when the funnel plot is symmetrical (i.e., no bias).

### Certainty Assessment

The results of the meta-analysis and risk of bias assessment were used to complete a Grading of Recommendations, Assessment, Development, and Evaluation (GRADE) certainty assessment [41]. Two researchers qualitatively assessed risk of bias, consistency and precision, and gave a summary rating – high, moderate or low certainty of evidence.

## Results

### Study Selection

Study selection results are presented in Fig. 1 (flow diagram). The search yielded a total of 2330 potentially relevant articles after the removal of duplicates. After reviewing titles and abstracts, we obtained and reviewed 212 full-text articles. Of these articles, 55 met the inclusion criteria. However, after contacting authors, two of these articles [42, 43] did not provide enough information to be included in the meta-analyses (*k* = 53).

### Study Characteristics

Articles were grouped by study design to align with the primary and secondary aims of the review. Study characteristics are detailed in OSM Resources 2 and 3. Publication dates ranged from 2009 to 2022. Most studies were published in Spain (*k* = 14), followed by the USA (*k* = 8), Australia (*k* = 6), Chile (*k* = 5), Taiwan (*k* = 3) and Finland (*k* = 3). Study designs were cross-sectional (*k* = 36), longitudinal (*k* = 8) and experimental (*k* = 11).

There was a total of 1,917,659 participants. The primary meta-analysis of resistance training studies included 1,235 participants, with a mean age ranging from 8.8 to 16.4 years. The secondary meta-analysis of cross-sectional and longitudinal studies included 1,916,424 participants, with a mean age ranging from 5.8 to 18 years. Most participants were adolescents (10–18 years, *k* = 33), followed by both adolescents and children (5–18 years, *k* = 14) and children (5–9 years, *k* = 6).

Cognition was measured using tests of attention (*k* = 7), cognitive flexibility (*k* = 7), inhibitory control (*k* = 19), working memory (*k* = 11), planning (*k* = 2) and fluid intelligence (*k* = 9). Academic achievement was measured in mathematics (*k* = 20), languages (*k* = 16) and combined scores (*k* = 15). Only one measure of on-task behaviour was included (*k* = 1).

### Risk of Bias

The risk of bias within studies for resistance training is presented in OSM Resource 4. Studies were defined as having low (*k* = 5), some (*k* = 3) or high (*k* = 2) concerns. The risk of bias within studies for muscular fitness is presented in OSM Resource 5. All studies clearly defined their research questions, and only one study failed to clearly define the study population. Studies were categorized as poor (*k* = 1), fair (*k* = 21) or good (*k* = 23).

**Fig. 2:**
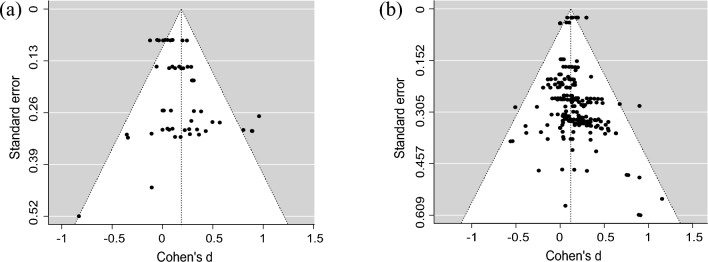
Funnel plots depicting publication bias: (**a**) resistance training studies; (**b**) muscular fitness studies

To determine if there was publication bias, funnel plots were created for the primary and secondary objectives (Fig. 2). For resistance training, some asymmetry of effect sizes in the funnel plot and a significant Egger’s regression test (*z* = 3.72, *P* < 0.001) indicated that there was some evidence of publication bias. Regarding muscular fitness, relative symmetry of effect sizes in the funnel plot and a non-significant Egger’s regression test (*z* = 1.21, *P* = 0.25) indicated that publication bias was not a major concern.

### Synthesis of Results

Resistance training had a small positive effect on overall cognition, academic performance and on-task behaviour (SMD 0.19, 95% CI 0.05–0.32) (Table 2). A minimal proportion of the variation within this pooled effect was attributable to differences within studies (${I}^{2}$ = 7%); however, differences between studies (${I}^{2}$ = 43%) may represent moderate heterogeneity.

**Table 2:** The effect of resistance training on cognitive and academic outcomes in school-aged youth

Variable	*k*	Number of effect sizes	Effect size (Cohen's *d)*	Lower 95% CI	Upper 95% CI	${I}^{2}\_2$	${I}^{2}\_3$	${\tau }^{2}\_2$	${\tau }^{2}\_3$	${R}^{2}\_2$	${{\varvec{R}}}^{2}\_3$
Overall academic, cognitive, and on-task behaviour performance	10	53	**0.19**	0.05	0.32	0.07	0.43	0.003	0.018		
Moderator analyses											
*Cognitive and academic outcomes*								0.003	0.012	0.000	0.361
Academic achievement—mathematics	2	2	0.24	− 0.03	0.51						
On− task behaviour	1	2	0.39	− 0.14	0.88						
Cognition	10	49	**0.17**	0.06	0.30						
*Aspects of cognition*								0.003	0.033	0.000	0.000
Working memory	3	15	0.13	− 0.07	0.29						
Inhibition	7	20	0.14	− 0.05	0.28						
Cognitive flexibility	3	7	0.06	− 0.20	0.30						
Attention	2	7	0.11	− 0.22	0.44						
*Participant characteristics*											
Age								0.003	0.018	0.003	0.010
Children 5–9 y	1	1	− 0.01	− 1.03	0.82						
Adolescents 10–18 y	9	52	**0.19**	0.06	0.32						
*Study characteristics*											
Study design								0.003	0.018	0.000	0.000
Acute	5	26	0.16	− 0.04	0.33						
Chronic	5	27	**0.22**	0.03	0.41						
Intervention type								0.003	0.011	0.000	0.403
Resistance training	5	20	**0.26**	0.10	0.42						
Concurrent training	5	33	0.11	− 0.05	0.28						
Risk of bias								0.003	0.019	0.005	0.000
Poor (high risk of bias)	2	13	**0.21**	0.04	0.40						
Fair (some concerns)	3	10	0.20	− 0.06	0.44						
Good (low risk of bias)	5	30	0.06	− 0.35	0.38						

The summary measures for resistance training and its effect on cognition and academic outcomes are in Cohen’s *d*. A Cohen’s *d* of 0.2 is interpreted as small, 0.5 represents medium, and 0.8 a large effect size

*I*^2^_2 = heterogeneity at Level 2 (i.e., between effect sizes from the same study); *I*^2^_3 = heterogeneity at Level 3 (i.e., between studies). $\tau$^2^_2 = within study variance; $\tau$^2^_3 = between study variance; *R*^2^_2 = variance explained at Level 2 (i.e., between effect sizes from the same study); *R*^2^_3 = variance explained at Level 3 (i.e., between studies).

Figures in bold represent effect sizes with confidence intervals that did not cross zero

The association between muscular fitness and cognition and academic achievement combined was positive but very small (SMD 0.13, 95% CI 0.10–0.16) (Table 3). Within this pooled effect size, heterogeneity within (${I}^{2}$ = 17%) and between (${I}^{2}$ = 5%) studies was negligible.

**Table 3:** The associations between muscular fitness and cognitive and academic outcomes in school aged youth

Variable	*k*	Number of effect sizes	Effect size (Cohen's *d)*	Lower 95% CI	Upper 95% CI	${I}^{2}\_2$	${I}^{2}\_3$	${\tau }^{2}\_2$	${\tau }^{2}\_3$	${R}^{2}\_2$	${R}^{2}\_3$
Overall academic and cognitive performance combined	43	211	**0.13**	0.10	0.16	0.17	0.05	0.003	0.001		
Moderator analyses											
*Cognitive and academic outcomes*								0.003	0.001	0.000	0.422
Overall cognition	24	113	**0.12**	0.07	0.17						
Overall academic achievement	28	98	**0.15**	0.09	0.18						
Aspects of cognition								0.003	0.007	0.000	0.000
Attention	5	10	0.08	− 0.10	0.26						
Inhibition	12	26	0.09	− 0.03	0.21						
Cognitive flexibility	4	18	**0.18**	0.03	0.32						
Working memory	8	26	**0.14**	0.02	0.26						
Planning	2	5	0.26	− 0.05	0.57						
Fluid intelligence	9	28	**0.11**	0.01	0.21						
School subject								0.002	0.007	0.213	0.000
Mathematics	18	39	**0.17**	0.08	0.25						
Languages	16	32	**0.10**	0.01	0.18						
Combined academics	15	27	**0.12**	0.02	0.22						
*Fitness*											
Data adjusted for cardiorespiratory fitness								0.003	0.000	0.039	0.000
No	36	172	**0.12**	0.07	0.16						
Yes	8	39	**0.15**	0.08	0.19						
*Participant characteristics*											
Age								0.003	0.011	0.005	0.00
Children 5–9 y	5	14	0.14	− 0.04	0.32						
Adolescents 10–18 y	24	115	**0.10**	0.03	0.16						
Both 5–18 y	14	82	**0.19**	0.12	0.26						
*Study characteristics*											
Study design								0.003	0.001	0.013	0.407
Cross-sectional	35	174	**0.14**	0.10	0.17						
Longitudinal	8	37	**0.09**	0.02	0.16						
Risk of bias								0.003	0.001	0.000	0.272
Poor (high risk of bias)	1	2	0.27	− 0.31	0.86						
Fair (some concerns)	19	90	**0.12**	0.07	0.18						
Good (low risk of bias)	23	119	**0.13**	0.09	0.17						

The summary measures for muscular fitness and its relationship with cognition and academic outcomes are in Cohen’s *d*. A Cohen’s *d* of 0.2 is interpreted as small, 0.5 represents medium and 0.8 a large effect size

*I*^2^_2 = heterogeneity at Level 2 (i.e., between effect sizes from the same study); *I*^2^_3 = heterogeneity at Level 3 (i.e., between studies). $\tau$ ^2^_2 = within study variance; $\tau$^2^_3 = between study variance; *R*^2^_2 = variance explained at Level 2 (i.e., between effect sizes from the same study); *R*^2^_3 = variance explained at Level 3 (i.e., between studies)

Figures in bold represent effect sizes with confidence intervals that did not cross zero

### Primary Outcome Moderator Analyses: Effect of Resistance Training on Cognitive and Academic Outcomes

#### Cognition and Academic Outcomes

Aspects of cognition moderated the effect of resistance training on overall cognitive, academic and on-task behaviour performance (${R}^{2}$ = 0.36). Resistance training had a small positive effect on cognition (SMD 0.17, 95% CI 0.06–0.30), academic achievement (SMD 0.24, 95% CI − 0.03 to 0.51), and on-task behaviour (SMD 0.39, 95% CI − 0.14 to 0.88); however, the confidence intervals for academic achievement and on-task behaviour crossed zero.

#### Participant Characteristics

Age did not moderate the effect of resistance training on academic outcomes (${R}^{2}$ = 0.01).

#### Study Characteristics

Study design (i.e., acute vs. chronic studies) did not have a moderating effect. However, intervention type moderated the effect of resistance training on overall cognition and academic outcomes (${R}^{2}$ = 0.40). Resistance training significantly improved cognition and academic outcomes (SMD 0.26, 95% CI 0.10–0.42), while the result for concurrent training was less effective (SMD 0.11, 95% CI − 0.05 to 0.28).

#### Risk of Bias

Risk of bias did not moderate the effect of resistance training on cognition and academic outcomes (${R}^{2}$ = 0.00).

### Secondary Outcome Moderator Analyses: Association Between Muscular Fitness and Cognition and Academic Outcomes

#### Cognition and Academic Outcomes

Aspects of cognition moderated the association between muscular fitness and overall cognition and academic outcomes (${R}^{2}$ = 0.42). Cognitive flexibility, working memory and fluid intelligence showed very small positive associations (SMD 0.18, 95% CI 0.03–0.32; SMD 0.14, 95% CI 0.02–0.26; SMD 0.11, 95% CI 0.01–0.21). The other aspects of cognitive function were not significantly associated with muscular fitness. In addition, we found a small positive association for studies that included mathematics (SMD 0.17, 95% CI 0.08–0.25), languages (SMD 0.10, 95% CI 0.01–0.18) and combined academic results (SMD 0.12, 95% CI 0.02–0.22).

#### Fitness

Adjustment for cardiorespiratory fitness did not moderate the association between muscular fitness and overall cognition and academic outcomes (${R}^{2}$ = 0.00).

#### Participant Characteristics

Age did not moderate the association between muscular fitness and overall academic and cognitive performance combined (${R}^{2}$ = 0.00). However, a small association was evident for studies across both age ranges (SMD 0.19, 95% CI 0.12–0.26) and negligible for adolescents (SMD 0.10, 95% CI 0.03–0.16).

#### Study Characteristics

Study design moderated the association between muscular fitness and cognition and academic outcomes (${R}^{2}$ = 0.41). The very small positive effect was marginally stronger for cross-sectional studies (SMD 0.14, 95% CI 0.10–0.17) than longitudinal studies (SMD 0.09, 95% CI 0.02–0.16).

#### Risk of Bias

Risk of bias within studies moderated the association between muscular fitness and cognition and academic outcomes (${R}^{2}$ = 0.27). Studies rated as fair or good reported very small positive results (SMD 0.12, 95% CI 0.07–0.18; SMD 0.13, 95% CI 0.09–0.17).

### Certainty Assessment

The certainty of evidence for the primary and secondary outcomes are displayed in Table 4.

**Table 4:** Certainty of evidence for the impacts of resistance training and associations of muscular fitness

Variable	#No. of studies	n	Findings	Certainty of evidence
Resistance training				
Overall academic, cognitive, and on− task behaviour performance	10	1235	SMD, 0.19 (0.05 to 0.32)	Low certainty
Muscular fitness				
Overall academic and cognitive performance	43	1,916,424	SMD, 0.12 (0.08 to 0.16)	Moderate certainty

*SMD* standardized mean difference

## Discussion

To our knowledge, this is the first systematic review and meta-analysis designed to investigate the effects of resistance training and muscular fitness on cognitive and academic outcomes in school− aged youth. Our primary outcome findings suggest that resistance training has a small positive effect on cognition and academic outcomes (low certainty evidence). Our meta-analysis of cross− sectional and longitudinal studies for our secondary outcome identified a small positive association between muscular fitness and cognition and academic achievement (moderate certainty evidence). Academic achievement, particularly in mathematics, was more strongly associated with muscular fitness than cognitive function. However, the inclusion of cross− sectional studies limited the heterogeneity so our findings should be interpreted with caution.

Evidence for the benefits of physical activity for young people’s cognition has been accumulating in recent years; however, most research has focused on aerobic exercise [7, 44–46]. Systematic reviews provide support for the use of aerobic training to improve cognitive and academic outcomes in children and adolescents [47–49], though other qualitative approaches including mindfulness practices or cognitive training are favored by some researchers [50]. Until now, information regarding the cognitive benefits of resistance training for youth has not been quantitatively synthesized.

Our moderator analyses revealed that resistance training interventions produced improvements in the combined aspects of cognition (SMD 0.17, 95% CI 0.06–0.30). Cognitive tasks of varying complexity were included in the analysis and our findings are consistent with those observed in a recent review, where overall exercise had a positive effect on all three core executive functions: working memory, inhibition, and cognitive flexibility [35]. The analysis of our included constructs (attention, cognitive flexibility, inhibition, working memory, planning, and fluid intelligence) revealed no specific construct of cognition, showed greater sensitivity to resistance training. Unfortunately, we were unable to analyze the effect of resistance training on academic achievement and on− task behaviour due to the limited number of studies. More research is warranted in this area so that future reviews can explore these factors in greater detail.

When comparing study characteristics, we found that acute and chronic studies produced similar effects. Long− standing evidence exists for the benefits of acute physical activity on cognition [35, 51], while, in contrast to our findings, chronic outcomes can be inconsistent [52, 53]. It is important to acknowledge that while the consequences of resistance training on overall cognition may be similar, the mechanisms involved are different. Despite the positive results shown for resistance training, further quantitative and qualitative factors should be considered. For example, the intensity, frequency [54] and demand of cognition [55] could also be taken into consideration, as well as the significant positive effect sometimes observed when interventions are led by highly qualified practitioners [54]. Nevertheless, this finding highlights the need for subsequent studies to deliver resistance training programs to youth that provide opportunities for acute and chronic responses through delivery in educational settings.

We found that resistance training resulted in larger improvements in cognition and academic outcomes than concurrent training. A moderate amount of variance was evident in this finding, which could possibly be attributed to the scope of the interventions delivered. Interestingly, most of the resistance training interventions included in our review were delivered in schools. Educational settings can be practical for targeting school− aged youth to increase physical activity, as students are required to spend most of the day in a classroom, where sedentary behaviours are prevalent. In this context, the inclusion of resistance training into active learning (e.g., classroom activity breaks, cognitively challenging physical activity) may therefore be warranted. Reviews have found positive effects for active learning on academic achievement but not executive function [56, 57]. Donnelly and Lambourne [58] discuss the challenges of designing classroom active breaks that require minimal disruption to the learning environment while providing adequate intensity and energy expenditure. Bodyweight exercises (e.g., squats) could be a feasible solution as they are a scalable form of resistance training that does not require equipment and is suitable for children and adolescents [3, 59, 60].

Although not examined in our review, it is interesting to consider the potential mechanisms responsible for the effect of resistance training on cognition. Lubans et al. [13] suggest three broad categories of mechanisms (i.e., neurobiological, psychosocial and behavioural) responsible for the effects of physical activity on cognition. Of relevance to our current review is the behavioural mechanism, as changes in coping and self− regulation skills may lead to improvements in executive function. In the school setting, on− task behaviour is seen as synonymous with self− regulation and, although limited by available studies, the results of our meta-analysis align with the evidence of behavioural mechanisms. The allocation of mental resources during exercise may also contribute to cognitive improvements [61]. Alternatively, resistance training is a motor coordinative task requiring focus and attention to safely execute each movement. The increased cognitive demand of resistance training may facilitate improvements in cognition and academic outcomes via neurogenesis [61]. Identifying the mechanisms that underlie the link between resistance training and improved cognition and academic outcomes for adolescents holds extraordinary promise for future research.

Despite limited evidence showing a link between muscular fitness and cognitive and academic outcomes, available data suggest there is an uncertain association for both cross− sectional and longitudinal studies [37]. In that regard, the large number of studies included in our meta-analysis offers a unique opportunity to further investigate the association between muscular fitness and cognitive function. It is important to note that studies assessing the association between muscular fitness and cognitive function were relatively consistent, with negligible heterogeneity. However, we acknowledge that there is considerable heterogeneity in the methods for assessing cognition and academic performance in the included studies.

The analysis of aspects of cognition was unable to explain any of the observed variance of the overall association of muscular fitness and cognition. Working memory, which allows for temporary storage and manipulation of information necessary for complex cognitive tasks, showed a strong association with muscular fitness [62]. Considered one of the core domains of executive function, improvements in working memory may benefit learning outcomes, and further, improvements in working memory may also benefit other higher level executive functions such as planning, reasoning and problem solving, leading to additional learning gains [63]. Comparative studies are not available for youth, but in a systematic review conducted in adults, Landrigan et al. [9] found that resistance training had little effect on working memory. This could suggest developmental changes due to age and maturity may make working memory more susceptible to resistance training effects. Cognitive flexibility (i.e., adjusting to new demands, rules or priorities) [63] and fluid intelligence (the ability to reason, problem solve, and to see patterns or relations among items) [64] had very small positive effects. All other constructs of cognition were not significantly associated with muscular fitness in our meta-analysis.

Mathematics showed the strongest relationship with muscular fitness, while languages and combined scores provided less evidence. This result is not unexpected given that complex mathematical concepts such as algebraic fractions have been shown to associate with working memory [65]. These results are supported by a recent systematic review from Santana et al. [37], which found positive associations between physical fitness and academic achievement. Of interest, the only study that was unable to be included in our meta-analysis also aligned with our findings, with Van Dunsen et al. [42] reporting that core strength (measured by curl− ups) was positively associated with overall academic performance. The practical application of these findings could inform future directions for the inclusion of muscle− strengthening activities to support student learning as mathematics/languages is embedded within most subjects in the school curriculum.

Participant age did not moderate the association between muscular fitness and cognition or academic achievement. Studies involving adolescents (10–18 years) and both age groups (5–18 years) provided the greatest indication of a positive association. The increased association for the wider age range may be attributed to the large number (92%) of included cross− sectional studies. Cross− sectional studies compared to longitudinal studies often report stronger evidence for a positive association between physical fitness and academic performance [37]. Our analysis of study design supports the findings of Santana et al. [37], with cross− sectional studies showing a stronger relationship between muscular fitness and cognition and academic achievement than longitudinal studies.

The meta-analysis for the benefits of resistance training and associations between muscular fitness and academic outcomes in youth provides further evidence for the benefits of muscle− strengthening activity, consistent with international guidelines. More specifically, our findings may assist researchers, policy makers and practitioners regarding the implementation of resistance training and muscular fitness. Where studies are currently having difficulty scaling up resistance training interventions in schools, our findings may offer support for their inclusion due to the benefits for young people’s cognitive and academic outcomes [66].

### Limitations

We identified only ten studies that had examined the impact of resistance training on cognitive and academic outcomes. Further, our meta-analysis consisted mostly of cross− sectional and longitudinal studies (*k* = 43). Some additional limitations include the following: first, cognition and academic performance were measured using a wide range of instruments that vary substantially in validity and reliability. Second, we combined standardized tests and school grades (evaluated by teachers) to assess academic achievement. Third, it is important to note the variability in resistance− training protocols included in our review. Due to the small number of studies, we were unable to compare the effects of different resistance− training protocols in terms of volume, intensity and type of training (e.g., free weights, body weight). Finally, there was some publication bias in the meta-analysis of resistance training and cognitive and academic outcomes due to inclusion of only published literature [67].

## Conclusions

Based on a limited number of studies we found select evidence that participation in resistance training has a small positive effect on cognition and academic outcomes in school− aged youth. Consistent with our findings from experimental studies, we found evidence for the benefit of muscular fitness for young people’s cognition and academic achievement. Our results suggest that including resistance training may help to improve cognition and academic achievement in school− aged youth.

### Supplementary Information

Below is the link to the electronic supplementary material.

Supplementary file1 (PDF 43 KB)

Supplementary file2 (PDF 121 KB)

Supplementary file3 (PDF 124 KB)

Supplementary file4 (PDF 62 KB)

Supplementary file5 (PDF 176 KB)

## Declarations

### Availability of Data and Material

All data relevant to the study are included in the article or uploaded as supplementary information.

### Code Availability

Code used during the meta-analysis is available on request from the corresponding author.
