# Supplementary material for: Effects of Resistance Training on Academic Outcomes in School-Aged Youth: A Systematic Review and Meta-Analysis
Source: Sports Med. 2023 Jul 19;53(11):2095–109. doi: 10.1007/s40279-023-01881-6 (PMC10587249; doi:10.1007/s40279-023-01881-6)
Supplement: Supplementary file 1 — Supplementary file1 (PDF 43 KB) [file 40279_2023_1881_MOESM1_ESM.pdf]

Online resource 1 Full search strategy

The selected search terms were:

("musc* strength" OR "musc* fitness" OR “resistance exercis*” OR "resistance training" OR “musc* power” OR “musc* endurance” OR "weight training" OR "weightlifting" OR "strength training") AND (cognition OR "academic achievement" OR "academic performance" OR "executive function" OR learning OR "on-task behav*" OR "on-task behav*" OR "student engagement" OR attention OR "goal-directed behav*" OR concentration OR “cognitive function” OR “cognitive control” OR memory OR inhibition OR “working memory” OR “task switching” OR “cognitive flexibility” OR “executive control” OR “mental flexibility”) AND (child* OR student* OR “school” OR adolescen* OR “preadolescen*” youth OR “young person” OR teen*).
