# Supplementary material for: Effects of Resistance Training on Academic Outcomes in School-Aged Youth: A Systematic Review and Meta-Analysis
Source: Sports Med. 2023 Jul 19;53(11):2095–109. doi: 10.1007/s40279-023-01881-6 (PMC10587249; doi:10.1007/s40279-023-01881-6)
Supplement: Supplementary file 2 — Supplementary file2 (PDF 121 KB) [file 40279_2023_1881_MOESM2_ESM.pdf]

Online resource 2 Key characteristics of studies examining the effect of resistance training on cognition and academic outcomes.

Author (year) Country	Study design	Sample size	Age (mean)	Control group / comparator	Duration	Frequency	Intervention description	Type	Cognition outcome/s	Outcome method of measurement	Acute/ Chronic
Costigan (2016) Australia	Individually-randomized parallel-group trial	n = NR (m)<br>n = NR (f)<br>N = 44 (m/f)	15.8 years	No exercise	8 weeks	3 x 10 minute sessions per week<br><br>Total 24 sessions	HIIT<br>50% CVT,<br>50% RT	CT	Cognitive flexibility	Trail making test	Chronic
Han-Byul (2015) Korea	Individually-randomized parallel-group trial	n = 8 (m)<br>n = 12 (f)<br>N = 20 (m/f)	8.75 years	No exercise	16 weeks	2 x 90 minute sessions per week<br><br>Total 32 sessions	Warm up, RT, CVT, Cool down	CT	Inhibitory control	Stroop test	Chronic
Harveson (2016) USA	Individually randomized cross-over trial	n = 48 (m)<br>n = 46 (f)<br>N = 94 (m/f)	(m)16.01 years<br>(f)16.14 years	No exercise	1 session	30 minutes	Weight machines<br>2 x 15 reps<br>6 exercises	RT	Inhibitory control<br>Cognitive flexibility	Stroop dot, Stroop colour, Stroop word.<br>Trail making test	Acute
Harveson (2018) USA	Individually randomized cross-over trial	n = 63(m)<br>n = 28 (f)<br>N = 91 (m/f)	15.89 years	No exercise	1 session	30 minutes	Weight machines<br>2 x 15 reps<br>6 exercises	RT	Maths<br>Inhibitory control	10-question math tests<br>Stroop dot, Stroop colour, Stroop word.	Acute
Harveson (2019) USA	Individually randomized cross-over trial	n = 57 (m)<br>n = 6 (f)<br>N = 63 (m/f)	13.7 years	No exercise	1 session	20 minutes	Weight machines<br>2 x 15 reps<br>6 exercises	RT	Maths<br>Inhibitory control	10-question math tests<br>Stroop dot, Stroop colour, Stroop word.	Acute
Leahy (2020) Australia	Cluster-randomized parallel-group trial	n = 32 (m)<br>n = 30 (f)<br>N = 62 (m/f)	16.2 years	No exercise	14 weeks	3 x 12-20 min<br><br>Total 42 sessions	HIIT<br>50% CVT,<br>50% RT	CT	Inhibitory control<br>Working memory	Modified flanker task<br>Serial n-back task	Chronic
Lubans (2020) Australia	Cluster-randomized parallel-group trial	n = 371 (m)<br>n = 299 (f)<br>N = 670 (m/f)	16.0 years	No exercise	1 year	2 x 8-20 minute teacher led session each week for 10 weeks	HIIT<br>50% CVT,<br>50% RT	CT	Inhibitory control<br>Working memory	Eriksen Flanker<br>Serial n-back task	Chronic

						Student then encouraged to complete sessions in their own time.					
Robinson (2021) Australia	Cluster-randomized parallel-group trial	n = 45 (m)<br>n = 31 (f)<br>N = 76 (m/f)	15.78 years	No exercise	4 weeks	3 x 8 min<br><br>Total 12 session	Tabata program of body weight resistance training in classroom	RT	Inhibitory control<br>Cognitive flexibility<br><br>On-task behaviour	Eriksen Flanker<br>Dimensional Change Card Sort Test<br>Adapted momentary time sampling procedure	Chronic
Wade (2020) Newcastle	Cluster-randomized parallel-group trial	n = 52 (m)<br>n = 38 (f)<br>N = 90 (m/f)	14.3 years	No exercise	1 session only	21 minutes	Six circuit stations including aerobic and resistance training activities.	CT	Attention<br><br>Working memory	Rapid Visual Information Processing (RVP) test.<br>Spatial working memory test	Acute
Yargic (2020) Turkey	Experimental	N = 25 (f)	16.4 years	No control	1 session	90 minutes	Weightlifting session weights >70% RM	RT	Attention	Finger tapping tests	Acute

Abbreviations: RT = Resistance training, CT = Concurrent training, HIIT = High intensity interval training, CVT = Cardiovascular training
