# Supplementary material for: Effects of Resistance Training on Academic Outcomes in School-Aged Youth: A Systematic Review and Meta-Analysis
Source: Sports Med. 2023 Jul 19;53(11):2095–109. doi: 10.1007/s40279-023-01881-6 (PMC10587249; doi:10.1007/s40279-023-01881-6)
Supplement: Supplementary file 3 — Supplementary file3 (PDF 124 KB) [file 40279_2023_1881_MOESM3_ESM.pdf]

Online resource 3 Key characteristics of studies examining the association between muscular fitness and cognition and academic outcomes

**Author (year) Country**	**Study design**	**Sample size**	**Age (mean)**	**Measure of MF**	**Test of MF**	**Measure of cognition**	**Test of cognition**	**Adjusted for CRF**
Aberg (2009) Sweden	Cross-sectional	N = 1221727 (m)	18 years	Combined	Isometric elbow flexion, knee extension, HG strength test	Cognition<br>Fluid intelligence	Four cognitive tests: Performance on all 4 tests were combined to obtain a global intelligence score	NO
Adelantado -Renau (2018) Spain	Cross-sectional	n = 138 (m)<br>n = 125 (f)<br>N = 263 (m/f)	13.9 years	Upper body<br>Lower body	HG strength test<br>SLJ test	Cognition<br>Fluid intelligence<br>Academic achievement<br>Combined	Questionnaire<br><br>GPA	NO
Aguilar (2015) Chile	Cross-sectional	n = 196 (m)<br>n = 196 (f)<br>N = 395 (m/f)	12.1 years	Lower body	SLJ test	Academic achievement<br>Combined<br>Maths<br>Languages	GPA<br>School grades for mathematics and language	NO
Amenya (2021) Ghana	Cross-sectional	n = 266 (m)<br>n = 325 (f)<br>N = 591 (m/f)	8 to 13 years	Upper body<br>Lower body<br>Core	HG strength test<br>SLJ test<br>SU test	Cognition<br>Fluid intelligence	Raven Coloured Progressive Matrices	NO
Bass (2013) USA	Cross-sectional	n = 395 (m)<br>n = 443 (f)<br>N = 838 (m/f)	13.1 years	Upper body<br>Core	Push Up test,<br>SU test	Academic achievement<br>Combined<br>Maths<br>Languages	Illinois Standardized Achievement Test (ISAT)<br>Maths and reading separated	NO
Cadenas-Sanchez (2017) Europe	Cross-sectional	n = 211 (m)<br>n = 233 (f)<br>N = 444 (m/f)	14.5 years	Upper body<br>Lower body	HG strength test<br>SLJ test	Cognition<br>Attention	d2 test	YES
Cadenas-Sanchez (2020) Spain	Cross-sectional	n = 61 (m)<br>n = 45 (f)<br>N = 106 (m/f)	10 years	Upper body<br>Lower body	HG strength test,<br>SLJ test, 1 RM in lab	Cognition<br>Fluid intelligence<br>Academic achievement<br>Combined	Woodcock-Muñoz test battery<br><br>GPA	YES
Cancela (2019) Spain	Cross-sectional	n = 336 (m)<br>n = 374 (f)<br>N = 713 (m/f)	14.2 years	Lower body	SLJ test, 30 sec chair stand test	Cognition<br>Inhibitory control<br>Working memory<br>Cognitive flexibility<br>Academic achievement<br>Combined	Stroop<br>Visual Memory-Weschsler Memory Scale<br>TMT<br><br>School grades	NO
Chen (2013) Taiwan	Longitudinal	n = 352(m)<br>n = 317 (f)<br>N = 669 (m/f)	14.6 years	Core	SU test	Academic achievement<br>Combined	School grades	NO

Coe (2013)<br>USA	Cross-sectional	n = NA (m)<br>n = NA (f)<br>N = 1701 (m/f)<br><br>Third Grade (N=291)<br>Sixth Grade (N=706)<br>Ninth Grade (N=704)	Not reported	Upper body<br>Core	Push Up test<br>SU test	Academic achievement<br>Maths<br>Languages	Standardized test scores	NO
Contreras-Osorio (2022)<br>Chile	Cross-sectional	n = 46 (m)<br>n = 44 (f)<br>N = 90 (m/f)	11.45 years	Upper body<br>Lower body	HG strength test, SLJ test	Cognition<br>Cognitive flexibility<br><br>Planning<br>Inhibitory control	The ENFEN Battery<br>Verbal fluency test, modified trail making test<br>Modified Hanoi towers<br>Modified Stroop	NO
De Castro (2016)<br>Brazil	Cross-sectional	n = 183 (m)<br>n = 143 (f)<br>N = 326 (m/f)	17.1 years	Core	SU test	Academic achievement<br>Combined	School grades	NO
de Greeff (2014)<br>Netherlands	Cross-sectional	n = 286 (m)<br>n = 258 (f)<br>N = 544 (m/f)	8.0 years	Combined	SLJ test, SU test, HG strength test	Academic achievement<br>Maths<br>Languages	Standardized test scores	NO
Dubuc (2020)<br>Canada	Longitudinal	n = 70 (m)<br>n = 115 (f)<br>N = 185 (m/f)	13.1 years	Upper body<br>Core	Push Up test<br>SU test	Cognition<br>Inhibitory control<br>Academic achievement<br>Combined<br>Maths<br>Languages	Flanker<br><br>School grades	NO
Esteban-Cornejo (2014)<br>Spain	Cross-sectional	n = 1049 (m)<br>n = 989 (f)<br>N = 2038 (m/f)	10.2 years	Combined	SLJ test, HG strength test	Academic achievement<br>Combined<br>Maths<br>Languages	School grades	YES
Eveland-Sayers (2009)<br>USA	Cross-sectional	n = 61 (m)<br>n = 73 (f)<br>N = 134 (m/f)	9.7 years	Core	SU test	Academic achievement<br>Maths<br>Languages	Standardized test scores	NO
Fochesatto (2022)<br>Brazil	Cross-sectional	n = 165 (m)<br>n = 152 (f)<br>N = 317 (m/f)	8 to 13 years	Upper body<br>Lower body<br>Core	SLJ test<br>2kg Med ball throw<br>SU test	Cognition<br>Fluid intelligence	Raven's coloured progressive matrix test	NO
García-Hermoso (2017)<br>Chile	Cross-sectional	n = 19,827 (m)<br>n = 17,043 (f)<br>N = 36,870 (m/f)	13.8 years	Lower body	SLJ test	Academic achievement<br>Maths<br>Languages	Standardized test scores	YES

Garcia-Hermoso (2020) Chile	Cross-sectional	n = 69 (m)<br>n = 132 (f)<br>N = 201 (m/f)	12.1 years	Combined	SLJ test, HG strength test	Cognition<br>Attention	 d2 test	NO
Gil-Espinosa (2019) Spain	Longitudinal	n = 112 (m)<br>n = 82 (f)<br>N = 194 (m/f)	14.15 years	Lower body	SLJ test	Academic achievement<br>Combined<br>Maths<br>Languages	 School grades	NO
Gil-Espinosa (2020) Spain	Cross-sectional	n = 216 (m)<br>n = 187 (f)<br>N = 403 (m/f)	13.7 years	Lower body	SLJ test	Cognition<br>Fluid intelligence<br>Academic achievement<br>Combined<br>Maths<br>Languages	D48 and Raven's Progressive Matrices.<br><br>School grades	NO
Haapala (2015) Finland	Cross-sectional	n = 202 (m)<br>n = 201 (f)<br>N = 403 (m/f)	7.6 years	Upper body<br>Core<br>Lower body	HG strength test<br>SU test<br>SLJ test	Cognition<br>Fluid intelligence	 Raven Coloured Progressive Matrices	NO
Hermassi (2021) Qatar	Cross-sectional	n = 36 (m)<br>n = 0 (f)<br>N = 36 (m/f)	9.0 years	Upper body<br>Lower body	Med ball throw<br>Squat jump and counter-movement jumps	Academic achievement<br>Maths	 School grades	NO
Haverkamp (2021) Netherlands	Cross-sectional	n = 199 (m)<br>n = 224 (f)<br>N = 423 (m/f)	13.5 years	Combined	SLJ and SU test combined	Cognition<br>Working memory<br>Inhibition<br>Attention<br>Academic achievement<br>Maths<br>Languages	 Grid test, Digit Span Task<br>Modified Attention Network test<br><br><br>Adapted standardized tests	NO
Hsieh (2018) Taiwan	Longitudinal	n = 198,774 (m)<br>n = 183,485 (f)<br>N = 382,259 (m/f)	Yr1 12.8 years<br>Yr3 14.8 years	Core<br>Lower body	SU test<br>SLJ test	Academic achievement<br>Combined	 Standardized test scores	NO
Kalantari (2016) Japan	Cross-sectional	n = 580 (m)<br>n = 0 (f)<br>N = 580 (m/f)	15.7 years	Upper body	Push Up test and HG strength test	Academic achievement<br>Combined	 School grades	NO
Kao (2017) USA	Cross-sectional	n = 44 (m)<br>n = 35(f)<br>N = 79 (m/f)	10.1 years	Combined	Full body battery of assessments	Cognition<br>Working memory<br>Academic achievement<br>Maths<br>Languages	 Serial n-back task<br><br>Adapted standardized tests	YES
Mora-Gonzalez (2019a) Spain	Cross-sectional	n = 58 (m)<br>n = 42 (f)<br>N = 100 (m/f)	10.1 years	Combined	SLJ test, and HG strength test	Cognition<br>Inhibitory control<br>Planning<br>Cognitive flexibility	 Stroop test<br>Zoo Map Test<br>Design Fluency Test and Trail Making Test	NO

Mora-Gonzalez (2019b)<br>Spain	Cross-sectional	n = 44 (m)<br>n = 35(f)<br>N = 79 (m/f)	10.2 years	Upper body<br>Lower body	HG strength test<br>SLJ test	Cognition<br>Working memory	Modified delayed non-matched-to-sample<br>Task (DNMS)	NO
Mora-Gonzalez (2020)<br>Spain	Cross-sectional	n = 47 (m)<br>n = 37 (f)<br>N = 84 (m/f)	10.1 years	Upper body<br>Lower body	HG strength test<br>SLJ test	Cognition<br>Inhibitory control	Flanker task	NO
Moradi (2019)<br>Iran	Cross-sectional	n = 206 (m)<br>n = 0 (f)<br>N = 206 (m/f)	11.0 years	Combined	SU test, and HG strength test	Cognition<br>Inhibitory control<br>Attention	Simon-task<br>Simple and 4-choice reaction time	NO
Muntaner-Mas (2018)<br>Spain	Cross-sectional	n = NA (m)<br>n = NA (f)<br>N = 234 (m/f)	10.98 years	Combined	SLJ test, and HG strength test	Academic achievement<br>Combined<br>Maths<br>Languages	School grades	YES
Muntaner-Mas (2021)<br>Spain	Longitudinal	n = 212 (m)<br>n = 210 (f)<br>N = 422 (m/f)	13.35 years	Combined	SLJ test, and HG strength test	Cognition<br>Working memory<br>Inhibition	Serial n-back task<br>Go/ no-go task	NO
Padulo (2019)<br>Italy	Cross-sectional	n = 34 (m)<br>n = 46 (f)<br>N = 80 (m/f)	11.0 years	Upper body<br>Lower body	Med ball throw<br>SLJ test	Academic achievement<br>Maths<br>Languages	School grades	NO
Ruiz (2010)<br>Spain	Cross-sectional	n = 868 (m)<br>n = 958 (f)<br>N = 1820 (m/f)	13.0 to 18 years	Lower body	SLJ test	Academic achievement<br>Maths	Standardized test	NO
Ruiz-Hermosa, (2020)<br>Spain	Cross-sectional	n = 304 (m)<br>n = 326 (f)<br>N = 630 (m/f)	5.8 years	Lower body	SLJ test	Academic achievement<br>Maths<br>Languages<br>Cognition<br>Fluid intelligence	Battery of General and Differential Aptitudes (BADyG E1)	NO
Ryu (2021)<br>South Korea	Cross-sectional	n = 60 (m)<br>n = 50 (f)<br>N = 110 (m/f)	9.0 years	Upper body<br>Lower body<br>Core	HG strength test<br>SLJ test<br>SU test	Academic achievement<br>Maths<br>Languages<br>Combined	School grades	NO
Sember (2022)<br>Slovenia	Longitudinal	n = 120 (m)<br>n = 111 (f)<br>N = 231(m/f)	Yr1, 11.3 years<br>Yr3, 14.3 years	Upper body<br>Lower body<br>Core	HG strength test<br>SLJ test<br>SU test	Academic achievement<br>Maths	School grades	
Shigeta (2021)<br>Australia	Cross-sectional	n = 306 (m)<br>n = 235 (f)<br>N = 541 (m/f)	16.5 years	Combined	Push Up test, and SLJ test	Cognition<br>Working memory<br>Inhibitory control	Serial n-back task<br>modified flanker task	YES

Solis-Urra (2021)<br>Chile	Cross-sectional	n = 591 (m)<br>n = 580 (f)<br>N = 1171 (m/f)	12.23 years	Combined	SLJ test, and HG strength test	Cognition<br>Cognitive flexibility<br><br>Working memory<br><br>Inhibitory control<br>Fluid intelligence	 Trail making tests<br>Digit symbol coding<br>Forward memory span<br>Reverse memory span<br>Go/ no-go task<br>Balance scale<br>Progressive matrices	NO
Syväoja (2019)<br>Finland	Longitudinal	n = 458 (m)<br>n = 496 (f)<br>N = 954 (m/f)	12.5 years	Combined	Push Up test, and SU test	Academic achievement<br>Combined	 School grades	NO
Syväoja (2021)<br>Finland	Cross-sectional	n = 127 (m)<br>n = 184 (f)<br>N = 311 (m/f)	14.0 years	Combined	Push Up test, and SU test	Cognition<br>Inhibitory control<br>Working memory<br><br>Attention<br><br>Academic achievement<br>Maths	 Flanker<br>Spatial Working Memory test, Digit span<br>Rapid Visual Information Processing<br><br>Basic arithmetic test, School grades	NO
Torrijos-Nino, (2014)<br>Spain	Cross-sectional	n = 183 (m)<br>n = 215 (f)<br>N = 398 (m/f)	9.49 years	Combined	SLJ test, and HG strength test	Academic achievement<br>Combined	 School grades	NO
Tsai (2016)<br>Taiwan	Cross-sectional	n = NA (m)<br>n = NA (f)<br>N = 80 (m/f)	8-12 years	Core<br>Lower body	SU test<br>SLJ test	Cognition<br>Inhibitory control	 Flanker test	NO
Van Dunsen (2011)<br>USA	Cross-sectional	n = 130684 (m)<br>n = 124059 (f)<br>N = 254743 (m/f)	Grades 3-11	Upper body<br>Core	Push Up test<br>SU test	Academic achievement<br>Languages<br>Maths	 Standardized test	NO

Abbreviations: MF = Muscular fitness, CRF = Cardiovascular fitness, HG = Hand grip, SLJ = Standing long jump, SU = Sit up
* = Not included in meta-analysis
