# Supplementary material for: Effects of Resistance Training on Academic Outcomes in School-Aged Youth: A Systematic Review and Meta-Analysis
Source: Sports Med. 2023 Jul 19;53(11):2095–109. doi: 10.1007/s40279-023-01881-6 (PMC10587249; doi:10.1007/s40279-023-01881-6)
Supplement: Supplementary file 4 — Supplementary file4 (PDF 62 KB) [file 40279_2023_1881_MOESM4_ESM.pdf]

Online resource 4 Risk of bias assessment checklist for resistance training studies (Cochrane risk-of-bias tool for randomized trials (RoB 2)).

	1. Randomization process	2. Deviations from intended interventions	3. Missing outcome data	4. Measurement of the outcome	5. Selection of the reported result	Overall score
Costigan (2016)	Low	Low	Low	Low	Low	Low
Han-Byul (2015	Some concerns	Low	Low	Low	Low	Some concerns
Harverson (2016)	Some concerns	Low	Some concerns	Low	Low	Some concerns
Harverson (2018)	Low	Low	Low	Low	Low	Low
Harverson (2019)	Low	Low	Some concerns	Low	Low	Some concerns
Leahy (2020)	Low	Low	Low	Low	Low	Low
Lubans (2020)	Low	Low	Low	Low	Low	Low
Robinson (2021)	Low	Low	Low	Low	Low	Low
Wade (2020)	Low	Low	Low	High	Low	High
Yargic (2019)	High	High	Low	Low	Low	High

Risk-of-bias judgment for each domain are: High = The study is judged to be at high risk of bias in at least one domain for this result, or the study is judged to have some concerns for multiple domains in a way that substantially lowers confidence in the result, Some concerns = the study is judged to raise some concerns in at least one domain for this result, but not to be at high risk of bias for any domain, Low = The study is judged to be at low risk of bias for all domains for this result.
