# Supplementary material for: Effects of Resistance Training on Academic Outcomes in School-Aged Youth: A Systematic Review and Meta-Analysis
Source: Sports Med. 2023 Jul 19;53(11):2095–109. doi: 10.1007/s40279-023-01881-6 (PMC10587249; doi:10.1007/s40279-023-01881-6)
Supplement: Supplementary file 5 — Supplementary file5 (PDF 176 KB) [file 40279_2023_1881_MOESM5_ESM.pdf]

Online resource 5 Risk of bias assessment checklist for muscular fitness studies (NIHM tool for observational studies)

	1. Research question	2. Study population	3. Participation rate	4. Participant exclusion and inclusion criteria	5. Sample size justified	6. Exposures (s) of interest were measured prior to the outcome(s) being measured	7. Sufficient time frame	8. The study examined different levels of the exposure as related to the outcome	9. Clarity of exposure measures	10. The exposure(s) was assessed more than once over time?	11. Clarity of dependent variable	12. Outcome assessors were blinded	13. Loss to follow-up after baseline was 20% or less	14. Were key potential confounding variables were addressed	Overall score
Aberg (2009)	Y	Y	Y	Y	Y	N	N	NR	Y	N	Y	NR	NR	N	FAIR
Adelantado-Renau (2018)	Y	Y	Y	Y	NR	N	N	Y	Y	N	CD	NR	Y	Y	FAIR
Aguilar (2015)	Y	Y	Y	Y	N	N	N	Y	Y	N	CD	NR	Y	Y	FAIR
Amenya (2021)	Y	Y	Y	Y	Y	N	N	Y	Y	N	Y	NR	Y	Y	GOOD
Bass (2013)	Y	Y	Y	Y	NR	N	N	Y	Y	N	CD	NR	Y	Y	FAIR
Cadenas-Sanchez (2017)	Y	Y	N	Y	NR	N	N	Y	Y	N	Y	NR	Y	Y	FAIR
Cadenas-Sanchez (2020)	Y	Y	Y	Y	N	N	N	Y	Y	N	Y	NR	Y	Y	GOOD
Cancela (2019)	Y	Y	Y	Y	Y	N	N	Y	Y	N	Y	NR	Y	Y	GOOD
Chen (2013)	Y	Y	Y	Y	NR	N	N	Y	Y	Y	CD	NR	Y	Y	GOOD
Coe (2013)	Y	Y	Y	Y	NR	N	N	Y	Y	N	Y	NR	Y	Y	GOOD
Contreras-Osorio (2022)	Y	Y	NR	Y	NR	N	N	Y	Y	N	Y	NR	Y	Y	FAIR
De Castro (2016)	Y	Y	Y	Y	Y	N	N	Y	Y	N	CD	NR	Y	Y	GOOD
de Greeff (2014)	Y	Y	NR	Y	NR	N	N	Y	Y	N	Y	NR	Y	Y	FAIR
Dubuc (2020)	Y	Y	N	Y	NR	N	N	Y	Y	Y	CD	NR	Y	Y	GOOD
Esteban-Cornejo (2014)	Y	Y	Y	Y	N	N	N	Y	Y	N	CD	NR	Y	Y	FAIR
Esteban-Cornejo (2017)	Y	Y	NR	Y	N	N	N	Y	Y	N	Y	NR	Y	Y	FAIR
Eveland-Sayers (2009)	Y	N	NR	Y	N	N	N	Y	N	N	Y	NR	Y	N	POOR

Fochesatto (2022)	Y	Y	NR	Y	Y	N	N	Y	Y	N	Y	NR	Y	Y	GOOD
Garcia-Hermoso (2016)	Y	Y	Y	Y	N	N	N	Y	Y	N	CD	NR	Y	Y	FAIR
Garcia-Hermoso (2017)	Y	Y	Y	Y	N	N	N	Y	Y	N	Y	NR	Y	Y	GOOD
Garcia-Hermoso (2020)	Y	Y	NR	CD	N	N	N	Y	Y	N	Y	NR	Y	Y	FAIR
Gil-Espinosa (2019) GENERAL INT	Y	Y	NR	Y	N	N	Y	Y	Y	N	Y	NR	NR	Y	FAIR
Gil-Espinosa (2019) PHYSICAL FIT	Y	Y	NR	Y	N	Y	Y	Y	Y	Y	CD	NR	NR	Y	GOOD
Gil-Espinosa (2020)	Y	Y	NR	Y	Y	N	N	Y	Y	N	Y	NR	Y	Y	GOOD
Haapala (2015)	Y	Y	Y	Y	N	N	N	Y	Y	N	Y	NR	Y	Y	GOOD
Hermassi (2021)	Y	Y	NR	Y	Y	N	N	Y	Y	N	CD	NR	Y	Y	FAIR
Haverkamp (2021)	Y	Y	NR	Y	N	N	N	N	Y	Y	Y	NR	Y	Y	
Hsieh (2018)	Y	Y	NR	Y	N	Y	Y	Y	Y	Y	Y	NR	Y	Y	GOOD
Kalantari (2016)	Y	Y	Y	Y	NR	N	N	Y	Y	N	CD	NR	N	Y	FAIR
Kao (2017)	Y	Y	Y	Y	N	N	N	Y	Y	N	Y	NR	Y	Y	GOOD
Mora-Gonzalez (2019) EXEC FUNC	Y	Y	Y	Y	NR	N	N	Y	Y	N	Y	NR	Y	Y	GOOD
Mora-Gonzalez (2019) NEUROELEC	Y	Y	Y	Y	NR	N	N	Y	Y	N	Y	NR	N	Y	FAIR
Mora-Gonzalez (2020) LOCALIZATI ON	Y	Y	Y	Y	NR	N	N	Y	Y	N	Y	NR	N	Y	FAIR
Mora-Gonzalez (2020) SEDENTARY	Y	Y	Y	Y	NR	N	N	Y	Y	N	Y	NR	N	Y	FAIR

Moradi (2019)	Y	Y	Y	Y	Y	N	N	Y	Y	N	Y	NR	Y	Y	GOOD
Muntaner-Mas (2018)	Y	Y	Y	Y	NR	N	N	Y	Y	N	CD	NR	Y	Y	FAIR
Muntaner-Mas (2022)	Y	Y	Y	Y	NR	N	N	Y	Y	N	Y	NR	Y	Y	GOOD
Padulo (2019)	Y	Y	Y	Y	Y	N	N	Y	Y	N	CD	NR	Y	Y	GOOD
Ruiz (2010)	Y	Y	Y	Y	NR	N	N	Y	Y	N	N	NR	Y	Y	FAIR
Ruiz-Hermosa (2020)	Y	Y	N	Y	NR	N	N	Y	Y	N	Y	NR	Y	Y	FAIR
*Ryu (2021)	Y	Y	Y	Y	NR	N	N	Y	Y	N	CD	NR	Y	Y	FAIR
Sember (2022)	Y	Y	Y	Y	NR	N	N	Y	Y	Y	CD	NR	Y	Y	FAIR
Shigeta (2021)	Y	Y	Y	Y	NR	N	N	Y	Y	N	Y	NR	Y	Y	GOOD
Solis-Urra (2021)	Y	Y	Y	Y	Y	N	N	Y	Y	N	Y	NR	Y	Y	GOOD
Syväoja (2019)	Y	Y	Y	Y	NR	N	Y	Y	Y	Y	CD	NR	Y	Y	GOOD
Syväoja (2021)	Y	Y	N	Y	NR	N	N	Y	Y	N	Y	NR	Y	Y	GOOD
Torrijos-Nino (2014)	Y	Y	Y	Y	NR	N	N	Y	Y	N	CD	NR	Y	Y	FAIR
Tsai (2017)	Y	Y	NR	Y	NR	N	N	Y	Y	N	Y	NR	Y	Y	FAIR
*Van Dunsen (2011)	Y	Y	N	N	N	N	N	Y	Y	N	Y	NR	Y	Y	FAIR

Y = yes; N = no; NA = not applicable; NR = not reported; CD = cannot determine. Overall scores were aligned with the Cochrane review: GOOD = low risk of bias; FAIR = some concerns; POOR = High risk of bias. * = Not included in meta-analysis
